# Predicting stability of DNA bulge at mononucleotide microsatellite

**DOI:** 10.1093/nar/gkab616

**Published:** 2021-07-26

**Authors:** Jin H Bae, David Yu Zhang

**Affiliations:** Department of Bioengineering, Rice University, Houston, TX 77005, USA; Department of Bioengineering, Rice University, Houston, TX 77005, USA; Systems, Synthetic, and Physical Biology, Rice University, Houston, TX 77005, USA

## Abstract

Mononucleotide microsatellites are clinically and forensically crucial DNA sequences due to their high mutability and abundance in the human genome. As a mutagenic intermediate of an indel in a microsatellite and a consequence of probe hybridization after such mutagenesis, a bulge with structural degeneracy sliding within a microsatellite is formed. Stability of such dynamic bulges, however, is still poorly understood despite their critical role in cancer genomics and neurological disease studies. In this paper, we have built a model that predicts the thermodynamics of a sliding bulge at a microsatellite. We first identified 40 common bulge states that can be assembled into any sliding bulges, and then characterized them with toehold exchange energy measurement and the partition function. Our model, which is the first to predict the free energy of sliding bulges with more than three repeats, can infer the stability penalty of a sliding bulge of any sequence and length with a median prediction error of 0.22 kcal/mol. Patterns from the prediction clearly explain landscapes of microsatellites observed in the literature, such as higher mutation rates of longer microsatellites and C/G microsatellites.

## INTRODUCTION

Microsatellites, which are also known as short tandem repeats of DNA, are clinically and forensically important. Due to their high mutability, microsatellites have been widely used as predictive (1–4), diagnostic (5–8), prognostic (9–12) and forensic biomarkers (13–16). They also act as markers in population studies (17–20) and have diverse functional roles (21). When genetic hypermutability is caused by an impaired DNA mismatch repair system, a condition called microsatellite instability (MSI) arises, where the number of tandem repeats grows or shrinks (22). This emphasizes the role of MSI as a molecular phenotype of tumor, but it also promotes oncogenesis (23).

**Figure 1.**
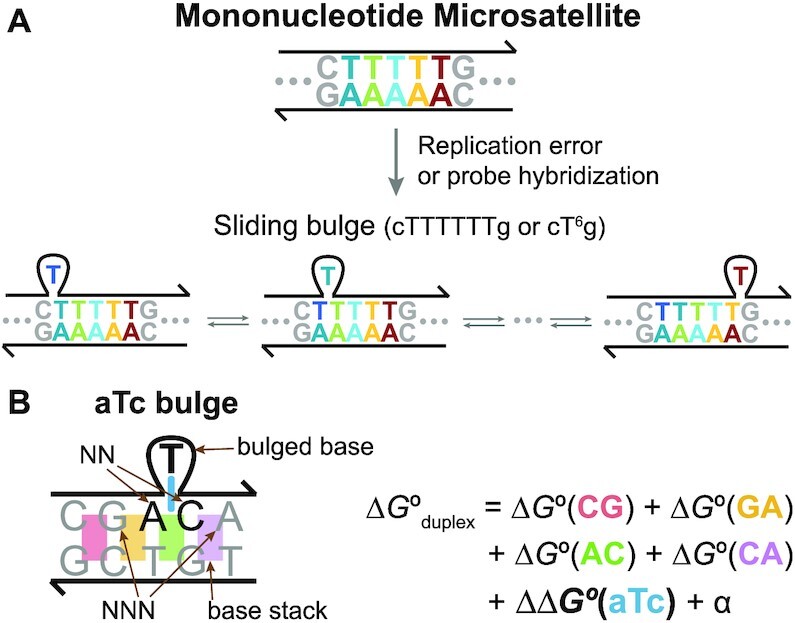
**(A)** A mononucleotide microsatellite is hypermutable short tandem repeats of a single base. When an extra T is added in this example, a bulged base is formed that can replace a neighboring T, causing a domino effect of sliding. We named this laterally sliding motif a sliding bulge. **(B)** Bulge thermodynamics based on the nearest-neighbor (NN) model. The name aTc bulge indicates that the bulged base is T, and its NNs are A and C. The bases next to the NN are called the next-nearest neighbors (NNNs). $\Delta G ^\circ _{\text{duplex}}$ is calculated as a sum of Δ*G*° of its base stacks (colored rectangles), a destabilizing ΔΔ*G*° of a bulge (blue rod) and a few constant terms.

In order for natural mutagenesis at a microsatellite to initiate, a strand slippage error occurs first during replication (24) to result in a special bulge with structural degeneracy (Figure 1A). Moreover, such bulges can be formed again when a hybridization probe or a PCR primer binds to a microsatellite with such mutation. We named this laterally sliding DNA motif a sliding bulge to distinguish from other static bulges. Characterization of sliding bulges is necessary to design effective probes and primers, understand how MSI occurs and, consequently, better exploit microsatellites as biomarkers. Because microsatellites in human genome are primarily mononucleotides (25) that go up to 83 repeats according to the reference genome assembly (GRCh38/hg38), the scope of this study is set to sliding bulges at mononucleotide microsatellites.

Despite the importance of sliding bulges, thermodynamics behind their stability is still poorly understood. One reason why there has been no systematic study is perhaps that various lengths of microsatellites give rise to a huge number of possible sliding bulges to be characterized. According to the nearest-neighbor (NN) model (26), which is the currently accepted DNA thermodynamic approximation, even the number of sliding bulges shorter than 30 tandem repeats is already over 1000. Another reason may be a lack of an accurate method for distinguishing small energy differences among many sliding bulges. Errors of DNA melting analysis were too significant (27) to confidently analyze the delicate thermodynamics of sliding bulges owing to structural degeneracy.

Here, we describe how we constructed and validated a predictive model of the thermodynamics of sliding bulges with any length and sequence. Toehold exchange energy measurement (TEEM) (28), which utilizes toehold exchange reactions in parallel to infer ΔΔ*G*° over a range of temperature independently (Supplementary Section S1), was used to accurately measure the free energy of bulges. We first resolved sequence-specific effects to systematically study destabilization of sliding bulges, and then observed how structural degeneracy affects destabilization. Based on the partition function (29), we characterized 40 common bulge states that can be assembled to any sliding bulge. We successfully validated our model by comparing it to experimental results and the literature.

## MATERIALS AND METHODS

### Materials

Phosphate-buffered saline (PBS, pH 7.4), Tris–EDTA (TE) and Tween 20 were purchased from Sigma-Aldrich. All oligonucleotides (oligos) were synthesized at the 100 nmol scale, dissolved in TE buffer (pH 8.0) to 100 μM and HPLC-purified by Integrated DNA Technologies (IDT). Chemical modifications on oligos were prepared by IDT as well. The concentrations of oligo stocks were verified with Nanodrop (Thermo Fisher), and then diluted to 10 μM in PBS. All oligos were stored in darkness at 4°C. The sequences of all oligos are listed in Supplementary Table S1. Solution fluorescence for TEEM was measured using a QuantStudio 7 Flex instrument (Applied Biosystems). Samples were loaded in MicroAmp Fast Optical 96-Well Reaction Plates, 0.1 ml (Applied Biosystems), and the loaded plate was sealed using MicroAmp Optical Adhesive Film (Applied Biosystems).

### TEEM

TEEM is described in detail and validated thoroughly in our previous publication (28). Briefly, it utilizes a toehold exchange reaction of C, P and X oligos, where both P and X oligos can hybridize with C oligo (Supplementary Section S1). Because P and X oligos are shorter than C oligo and aligned to its opposite ends, they can displace each other from C oligo. As a result, CP and CX duplexes exist in equilibrium according to their Δ*G*°, and we can calculate the free energy by measuring the concentrations of the duplexes. In order to infer the concentrations, we designed only CX duplex to emit fluorescence by functionalizing C and P oligos with a ROX fluorophore and an Iowa Black RQ quencher, respectively. Inferring ΔΔ*G*° of a motif requires toehold exchange reactions with X(reference) and its variation, X(bulge), oligos. The only difference between X(reference) and X(bulge) oligos is the presence of a bulge motif of interest, and subtracting their Δ*G*° of the reaction cancels everything out except ΔΔ*G*° of a bulge.

TEEM begins with diluting C, P, X(reference) and X(bulge) oligos from stock solutions to 0.5, 0.5, 0.4 and 10 μM, respectively, in PBS with 0.1% (v/v) Tween 20. C and P oligos were mixed first and then X(reference) oligo was added afterward to make their working concentrations 20, 30 and 40 nM, respectively. For reactions with X(bulge) oligos, their working concentration was 1 μM. Positive and negative control samples for characterizing maximum and minimum fluorescence signals were prepared by adding PBS in place of the P or X oligos, respectively.

To verify whether fluorescence measurements reflect actual equilibrium conditions, we measured fluorescence at every integer temperature between 20 and 70°C twice: once as the solution is being gradually cooled, and another time when the solution is being gradually warmed. Between the cooling and heating phases, temperature was maintained at 20°C for 1 h to check again whether equilibration is complete. The heating phase was the reverse of the cooling phase, and all temperature change was done at the rate of 2°C/s. Consistency between the two measurements implied that equilibrium was established.

## RESULTS

### Effect of the NNNs

The NN model approximates the free energy of hybridization $\Delta G ^\circ _{\text{duplex}}$ as a sum of their $\Delta G ^\circ _{\text{base stack}}$ and destabilization ΔΔ*G*° of a bulge as shown in Figure 1B. This bulge is called aTc bulge because AC base stack was disturbed by a bulged T, and now A and C are the NNs of the bulge. Using their relationship, bulge destabilization energy, or thermodynamic penalty, can be calculated as a Δ*G*° difference between a bulged duplex and its corresponding canonical duplex, hence ΔΔ*G*°.

**Figure 2.**
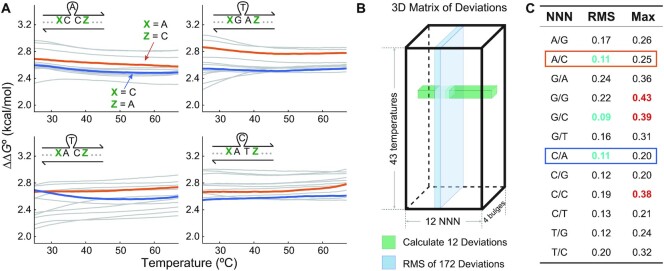
Process of selecting the representative NNNs for consistency in ΔΔ*G*° measurement. **(A)** ΔΔ*G*° of four non-slide bulges, each with 12 different NNNs (denoted as X and Z) at 43 different temperatures, were measured by TEEM (2064 ΔΔ*G*° values). After (B) and (C), A/C (orange) and C/A (dark blue) were to be selected as the representative NNNs for their best representativeness. **(B)** To find the NNNs that represent the others best, we compared ΔΔ*G*° values of each bulge with 12 different NNNs. A deviation of each NNN is defined as a difference between each ΔΔ*G*° and a mean of 12 ΔΔ*G*° values (green row). To evaluate overall representativeness of each NNN, a root mean square (RMS) of 172 deviations (blue plane) was calculated. A lower RMS means better representativeness. **(C)** As shown in (A), we selected two NNNs, A/C and C/A, with low RMS (teal) and without high maximum deviations (red).

To systematically measure ΔΔ*G*° of bulges, we first supplemented the NN model by reducing sequence-specific effects of local context. Although the thermodynamics of a perfectly matched duplex can be approximated well by considering only NNs, the same cannot be assumed for non-canonical structures like bulges. A bulge can disrupt a local double-helix structure (30), and this destabilization may reach bases beyond NNs. We thus defined NNNs as bases adjacent to NNs of a bulged base, and checked how NNNs affect bulge ΔΔ*G*° by measuring ΔΔ*G*° of four bulges (cAc, gTa, aTc and aCt) 12 times, each with different NNNs (Figure 2A). The measurement was performed at 43 different temperatures from 25 to 67°C, resulting in 2064 ΔΔ*G*° values in total. As expected, different NNNs showed different effects on bulge ΔΔ*G*°.

To keep consistency in ΔΔ*G*° measurement, we decided to select the representative NNNs and use them throughout this work. With a given NN and a bulged base, the representative NNNs should result in bulge ΔΔ*G*° close to a mean ΔΔ*G*° of all 12 NNNs. We introduce the concepts of a NNN deviation and an RMS of deviations to find NNNs with the highest representativeness across all temperatures and four bulges. An NNN deviation is defined as a difference between a ΔΔ*G*° value and a mean of 12 ΔΔ*G*° values from different NNNs. Because we tested four bulges at 43 temperatures, each NNN had 172 deviation values in total (Figure 2B), and RMS of each NNN evaluated the representativeness. Naturally, a lower RMS means better representativeness across different temperatures and bulge motifs. Because NNNs with low RMS could still have several huge deviations that damage the systematic approach, we also used maximum deviation as a secondary criterion to avoid any extreme cases. By picking three NNNs with the lowest RMS and avoiding three NNNs with the highest maximum deviations, A/C (A and C next to 5′ and 3′ ends of the bulge NN, respectively) and C/A were selected as the representative NNNs to measure ΔΔ*G*° twice and take an average for further experiments (Figure 2C).

### Bulge ΔΔ*G*° measurement

**Figure 3.**
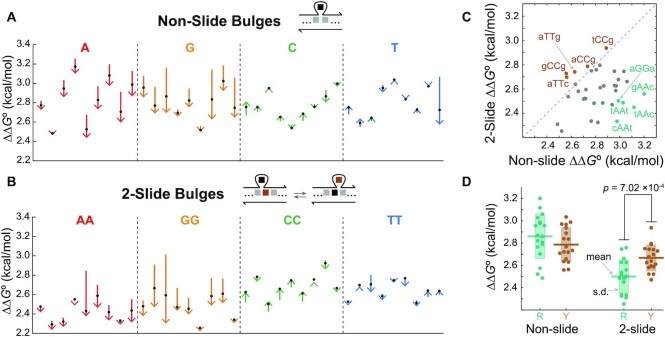
**(A)** ΔΔ*G*° of all 36 non-slide bulges at 43 different temperatures were measured as the reference and summarized by arrows. Each arrow starts at $\Delta \Delta G ^\circ _{25 ^\circ \text{C}}$ and ends at $\Delta \Delta G ^\circ _{65 ^\circ \text{C}}$ with each black dot indicating $\Delta \Delta G ^\circ _{45 ^\circ \text{C}}$. Bulges with the same bulged base are organized in alphabetical order. **(B)** Summarized ΔΔ*G*° of all two-slide bulges. **(C)** ΔΔ*G*° of two-slide bulges plotted against ΔΔ*G*° of the corresponding non-slide bulges at 37°C. ΔΔ*G*° of a two-slide bulge is generally lower than that of a non-slide bulge. The exceptions are colored in brown, which all have the pyrimidine bases (C and T) as bulges, whereas the examples in the opposite end (teal) have the purine bases (A and G). **(D)**$\Delta \Delta G ^\circ _{37 ^\circ \text{C}}$ grouped by ring types of a bulged base. R and Y denote purine and pyrimidine, respectively. Only two-slide bulges show a statistically significant difference (Welch’s *t*-test) between purine and pyrimidine, which explains the polarized result in (C).

Using the selected representative NNNs, we investigated how structural degeneracy of a sliding bulge affects the thermodynamics. As the reference, ΔΔ*G*° of all 36 bulges with a single dominant state and no sliding (non-slide bulge) were first measured at 43 different temperatures from 25 to 67°C. There are 36 bulges because a bulged base can be any of four bases, while the nearest non-bulged bases have to be different from the bulged base, leaving them three choices each. Figure 3A summarizes the results with each arrow starting at $\Delta \Delta G ^\circ _{25 ^\circ \text{C}}$ and ending at $\Delta \Delta G ^\circ _{65 ^\circ \text{C}}$ of a bulge, and black dots on the arrows indicate $\Delta \Delta G ^\circ _{45 ^\circ \text{C}}$. Complete datasets with all ΔΔ*G*° values are provided in Supplementary Section S2. We grouped the bulges by their bulged bases to highlight differences in slopes among them, and there was no other grouping method that revealed other differences (Supplementary Table S2). It is notable that ΔΔ*G*° of bulges with purine bases (A and G) are generally affected more by temperature, shown by the longer arrows. Moreover, their directions are always downward, implying that they have lower thermodynamic penalties at higher temperature.

As a comparison, we then measured ΔΔ*G*° of two-slide bulges that have sliding between two degenerate states (Figure 3B). There are 36 two-slide bulges, which are formed when a bulged base and one of the neighboring bases are the same, allowing them to replace each other. In addition to having the same trends observed in non-slide bulges, two-slide bulges with a purine base showed slightly lower ΔΔ*G*° than pyrimidine bulges (C and T) in this dataset. Both non- and two-slide bulges generally showed lower ΔΔ*G*° than their RNA counterparts (31,32), confirming that DNA bulges were less destabilizing. To observe the effect of structural degeneracy, ΔΔ*G*° of two-slide bulges were plotted against those of their corresponding non-slide bulges (e.g. aTc versus aTTc) at 37°C (Figure 3C). Most of 36 bulge pairs were below the diagonal line with five exceptions (brown), implying that the degeneracy generally decreased ΔΔ*G*° and stabilized two-slide bulges.

Interestingly, all five exceptions have a pyrimidine base as a bulged base, whereas the motifs on the opposite side (teal) have a purine base. This polarized result between pyrimidine and purine bulges can be explained when $\Delta \Delta G ^\circ _{37 ^\circ \text{C}}$ values of each motif are grouped by the ring types (Figure 3D). While ΔΔ*G*° of both purine and pyrimidine bulges were decreased by structural degeneracy, the change was more significant for purine bulges. Welch’s unequal variance *t*-test results suggest that non-slide bulges do not show any statistical difference between purine and pyrimidine bulges, but two-slide bulges do.

### Sliding bulge model construction

To build a predictive model of sliding bulge stability from the measured data, we laid groundwork of the model construction with the NN model of DNA thermodynamics. ΔΔ*G*°, which is a measure of destabilization, shows the ratio of the new equilibrium constant *K*_2_ to the old *K*_1_:

$$\begin{eqnarray*} \Delta \Delta G^{\circ } &=& \Delta G^{\circ }_{2}\: -\: G^{\circ }_{1} \\ &=& -RT \, \text{ln} \,K_{2} - (-RT \, \text{ln}\, K_{1} )= -RT \, \text{ln} \,\left(\frac{K_{2}}{K_{1}}\right). \end{eqnarray*}$$

**Figure 4.**
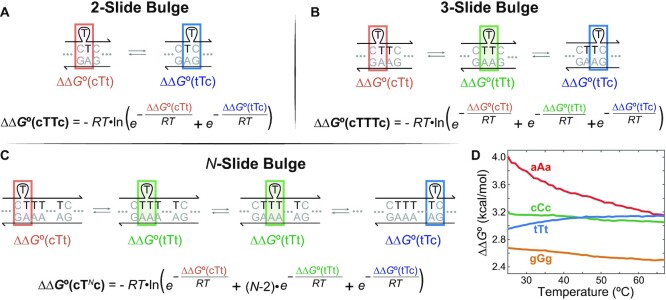
An example of a thermodynamic model of sliding bulges based on the partition function. **(A)** ΔΔ*G*° of a two-slide bulge cTTc can be expressed by ΔΔ*G*° of two degenerate states (orange and blue boxes). **(B)** Compared to cTTc bulge, a three-slide bulge cTTTc has one more state (green box) that we named T triplet state. **(C)** Likewise, an *N*-slide bulge is formed when a bulged base and *N* − 1 of neighboring bases are identical. Because the only difference between cT^*N*^c and cTTc bulges is the number of T triplet states, inferring ΔΔ*G*°(tTt) enables the model to predict ΔΔ*G*°(cT^*N*^c) with any *N* value. T triplet state can be isolated by comparing ΔΔ*G*°(cTTc) and ΔΔ*G*°(cTTTc). **(D)** Inferred mean ΔΔ*G*° of each triplet state. Measured ΔΔ*G*° of three three-slide bulges used for the calculation are shown in Supplementary Section S3.

The partition function, which derives the thermodynamic properties of the equilibrium conformational ensemble (29), can be readily applied to predicting overall destabilization from degenerate states of a sliding bulge. Our model infers ΔΔ*G*° of a sliding bulge by combining ΔΔ*G*° of each degenerate bulge state into a partition function. Figure 4 shows examples of the energy calculation and how ΔΔ*G*°(cT^*N*^c) can be calculated from ΔΔ*G*°(cTTc) and ΔΔ*G*°(cTTTc). First, ΔΔ*G*° and the partition function *Z* of cTTc bulge are expressed with ΔΔ*G*° of its two states (Figure 4A):

$$\begin{eqnarray*} \Delta \Delta G ^\circ \text{(cTTc)} &=& -RT \, \text{ln} (Z) \\ &=& -RT \, \text{ln}\left({\rm e}^{-{\Delta \Delta G ^\circ ({{\rm cTt}})}/{RT}}+{\rm e}^{-{\Delta \Delta G ^\circ ({{\rm tTc}})}/{RT}}\right). \end{eqnarray*}$$

ΔΔ*G*°(cTTTc) can be expressed in a similar way (Figure 4B):

$$\begin{eqnarray*} &&\Delta \Delta G ^\circ \text{(cTTTc)} \\ &&\quad= -RT \, \text{ln}\left({\rm e}^{-{\Delta \Delta G ^\circ ({{\rm cTt}})}/{RT}}+{\rm e}^{-{\Delta \Delta G ^\circ ({{\rm tTc}})}/{RT}}+{\rm e}^{-{\Delta \Delta G ^\circ ({{\rm tTt}})}/{RT}}\right). \end{eqnarray*}$$

The only difference between ΔΔ*G*°(cTTc) and ΔΔ*G*°(cTTTc) is ΔΔ*G*°(tTt) term (green), which we named T triplet state, so it can be numerically separated from the equations (Supplementary Section S3).

T triplet state plays the key role in expanding the prediction to a longer sliding bulge cT^*N*^c. Using the separated ΔΔ*G*°(tTt), the following equation can be used to predict ΔΔ*G*°(cT^*N*^c) with any *N* value (Figure 4C):

$$\begin{eqnarray*} &&\Delta \Delta G ^\circ (\text{cT}^N\text{c})\\ &&\quad = -RT \, \text{ln} \left( {\rm e}^{-{\Delta \Delta G ^\circ ({{\rm cTTc}})}/{RT}}+{(N-2)} \, {\rm e}^{-{\Delta \Delta G ^\circ ({{\rm tTt}})}/{RT}} \right), \end{eqnarray*}$$

If we apply the same reasoning to other sequences, ΔΔ*G*° of 40 common bulge states, which are 36 two-slide bulges and 4 triplet states, work as common building blocks that can be assembled into ΔΔ*G*° of any sliding bulge.

With ΔΔ*G*° data of all two-slide bulges acquired, the only missing building blocks for the predictive model were ΔΔ*G*° of the triplet states. Because ΔΔ*G*° of each triplet state can be extracted from ΔΔ*G*° of a two-slide bulge and its corresponding three-slide bulge (Supplementary Section S3), we measured ΔΔ*G*° of three three-slide bulges each. They were lower than ΔΔ*G*° of two-slide bulges in general, and ΔΔ*G*° of the G bulges were especially lower than the others (Supplementary Section S2). Figure 4D shows ΔΔ*G*° of the triplet states calculated from the mean ${\rm e}^{-{\Delta \Delta G ^\circ ({{\rm triplet}})}/{RT}}$ of three three-slide bulges. ΔΔ*G*°(aAa) is much higher than ΔΔ*G*° of any two-slide bulge at lower temperature, suggesting it has a minor role in equilibrium conformational ensemble. However, as temperature goes up, ΔΔ*G*°(aAa) goes down, which makes ΔΔ*G*° of sliding A bulges more temperature dependent. In contrast, low ΔΔ*G*°(gGg) makes the partition function *Z* larger and overall ΔΔ*G*° smaller, especially when multiple G triplet states stack in longer bulges. This can be interpreted as more stable G triplet state contributing significantly to stabilizing sliding G bulges.

### Predicting thermodynamics of sliding bulges

**Figure 5.**
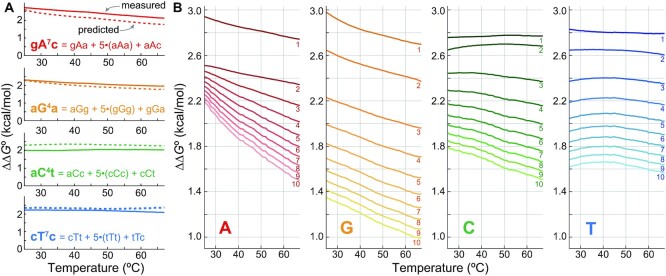
Validation and summary of sliding bulge ΔΔ*G*° predictions. **(A)** ΔΔ*G*° of four longer sliding bulges predicted by our model (solid lines) and experimentally measured by TEEM (dotted lines). A median and a maximum absolute value of residual were 0.22 and 0.37 kcal/mol, respectively. **(B)** Summary of predictions. Each line shows a mean of ΔΔ*G*° of all bulges with a given length and a bulged base, and a number next to each plot denotes the number of tandem repeats of a mononucleotide. Steep ΔΔ*G*° slopes of A bulges and low ΔΔ*G*° of G bulges reflect characteristics of their triplet states’ ΔΔ*G*°.

Using the predictive model based on the partition function and the ΔΔ*G*° data, we tested our prediction power by comparing predicted and measured ΔΔ*G*° values of four longer sliding bulges (Figure 5A). The lengths of C and G bulges were shorter than those of A and T bulges because sequences with long consecutive C or G had shown unreliable results under TEEM in our previous experience (28), which we attribute to secondary structures and limitations of oligo synthesis. A median and a maximum absolute value of residuals, which are differences between predicted and measured ΔΔ*G*°, were 0.22 and 0.37 kcal/mol, respectively. It is notable that temperature did not affect the residuals significantly because of the similar slopes between the predicted and the measured values.

As a quick reference, we plotted mean ΔΔ*G*° of all bulges with a given length and a bulged base (Figure 5B). A number next to each plot indicates the length of homopolymeric repeats. Sliding A bulges have steep ΔΔ*G*° slopes at lower temperature due to steep ΔΔ*G*°(aAa) slopes as displayed in Figure 4D, whereas ΔΔ*G*° of sliding G bulges have lower values because the low ΔΔ*G*°(gGg) contributes more to their stability. Of note, DNA with a long stretch of C or G may form secondary structures such as i-motif or G-quadruplex, resulting in deviations from the model. To make the prediction publicly available, we have created and attached a MATLAB function that only requires the sequence of a sliding bulge and temperature.

## DISCUSSION

In this work, we have built a model of sliding bulges at mononucleotide microsatellites to predict their ΔΔ*G*°. The model construction started with the theoretical work using the partition function to identify 40 common bulge states of sliding bulges, followed by careful ΔΔ*G*° measurements with TEEM. We first tested the effect of NNNs on bulge ΔΔ*G*° and selected the representative NNNs for the systematic study. Based on the groundwork, ΔΔ*G*° values of sliding bulges necessary to the model were measured.

Although Zhu and Wartell (31) did recognize structural degeneracy of a sliding bulge as the reason for its lower free energy, they failed to develop the observation into a proper model. After testing 10 sliding bulges, they used a single empirical constant to account for two- and three-slide bulges without realizing that the length of a sliding bulge affects ΔΔ*G*°. Thus, our model is the first to predict the free energy of longer sliding bulges, and it was based on the comprehensive analysis of the thermodynamics of sliding bulges. Moreover, our model construction principle can be further expanded to studying dinucleotide or trinucleotide sliding bulges that cause various neurological diseases (33–36).

With the theoretical background and the systematic data collection with TEEM, our model provides explanations to the experimental results of the literature on MSI. For example, researchers have observed that longer microsatellites tend to have higher mutation rates (37,38), which has been clearly elucidated by our thermodynamic model of sliding bulges based on the partition function. Our model also implies that sliding G bulges with the lowest penalty will dramatically stabilize longer microsatellites and drive mutation rates of C/G microsatellites up. Indeed, the longest C/G mononucleotide microsatellite from exome sequencing on 24 colorectal tumors was twice as long as the longest A/T mononucleotide microsatellite (81 bp versus 40 bp) (38). The same study revealed that the mutation rates of C/G microsatellites were 7.5 times higher on average than that of A/T when they had the same length and <10% margin of error. Other studies on MSI with human cancer cell lines reported similar results with C/G microsatellites showing 7 times (39) or 4.4 times (40) higher mutation rates.

In addition to offering theoretical explanations to landscapes of MSI, our model can help design experiments studying MSI. From a high-level point of view, the predictions made by the model can be used as a general guideline. Figure 5B shows how sequence and temperature affect ΔΔ*G*° of sliding bulges at a microsatellite, and such difference in penalty should be considered according to detail of an experiment. For instance, genotyping indels at poly(G) with a probe or a primer would be more difficult than genotyping poly(C) on the opposite strand due to low ΔΔ*G*° of sliding G bulges. And when probe hybridization protocol involves a temperature change due to washing steps, targeting poly(T) will be more consistent than targeting poly(A) because its ΔΔ*G*° is less dependent on temperature. The predicted ΔΔ*G*° values can also be used for fine-tuning probe or primer hybridization if more sophisticated approach is desired. By definition, adding ΔΔ*G*° of a sliding bulge to hybridization Δ*G*° of an original sequence without a sliding bulge gives the actual hybridization Δ*G*° of a sliding bulge formation. An equilibrium constant of hybridization can then be calculated from the resulting Δ*G*° and temperature, providing an estimation for a probe or a primer binding yield.

The purpose of the model construction was to predict ΔΔ*G*° of sliding bulges, but the non-slide bulge datasets acquired in the process are also useful by themselves. Those bulges can be easily formed during molecular biology experiments when primers or probes are hybridized with indel variants or non-specific targets. Thus, it is important to first predict DNA hybridization to properly design the experiments. There are a few data on the thermodynamics of non-slide bulges, but they were either a simple approximation with no experimental data (41) or measured at biologically irrelevant salinity with significant errors (42). In contrast, our non-slide bulge data from TEEM do not suffer from any of these problems (Supplementary Section S4) and can be readily applied to such predictions.

It is interesting that sliding bulges show clear differences according to whether they have a purine or a pyrimidine as a bulged base. As we constantly observed in non-, two- and three-slide bulges, ΔΔ*G*° of purine bulges decrease as temperature goes up. The temperature dependence of purine bulge ΔΔ*G*° implies that the entropy may be behind it. One possibility is that a bulkier purine base, which is a pyrimidine ring fused to an imidazole ring, causes more disorder when bulged out from a perfect double-helix structure. For example, larger purine bases could create larger hydration shells that limit mobility of water and, as a result, increase entropic impact. As a matter of fact, the literature on crystallographic data of unpaired RNA bases (43) and statistical analysis of hydration levels (44) showed purine bases had more water molecules around them than pyrimidine bases (Supplementary Table S3).

Another outcome of this work is a possibility of a sliding bulge that is more stable than a typical double-helix structure. In theory, a sliding bulge with enough tandem repeats could have so high structural degeneracy that its stabilizing effect overcomes thermodynamic penalty of having a bulge. Our model predicts that this singularity will happen when there are >41 G, >91 C, >101 A or >104 T repeats. Although such bulges will rarely appear *in vivo* or *in vitro* due to their lengths, their implication for a method of designing stable structures with structural degeneracy is intriguing.

An important limitation of our model is that errors from using the representative NNNs cannot be avoided. Because a bulge may disrupt a local helix structure, we used the representative NNNs throughout this work for consistency. This strategy effectively standardized bulge ΔΔ*G*° for the systematic model construction and prevented extreme ΔΔ*G*° deviation. However, some errors will always exist when the actual NNN is different from the representative NNNs unless we measure ΔΔ*G*° of every bulge with every NNN, which is impractical in terms of time and cost. In Figure 2A, the mean and the standard deviation of gaps between measured ΔΔ*G*° and their corresponding representative ΔΔ*G*° were 0.15 and 0.008 kcal/mol, respectively. See Supplementary Section S5 for a summary of all ΔΔ*G*° error values from the NNN choices. This error should be considered when the NNN is different from the representative NNNs used in this study.

## DATA AVAILABILITY

All experimental data are plotted in the Supplementary Materials, and numerical data files are available upon request.

## Supplementary Material

gkab616_Supplemental_Files
