# Supplementary material for: Predicting stability of DNA bulge at mononucleotide microsatellite
Source: Nucleic Acids Res. 2021 Jul 26;49(14):7901–8. doi: 10.1093/nar/gkab616 (PMC8373066; doi:10.1093/nar/gkab616)
Supplement: gkab616_Supplemental_Files [file gkab616_supplemental_files.zip › Supplementary Table legends.docx]

**Table S1**. Sequences of oligonucleotides used in each experiment

**Table S2.** Welch's unequal variances *t*-test results of non-slide bulge ∆∆*G*º and slopes. Those values were grouped by a base at 3'-end NN, 5'-end NN, or bulge, and then each group was compared to one another. Although all 3 criteria resulted in differences between some groups, only grouping by bulge base shows differences between all groups. Orange and red indicate *p*-values less than 0.05 and 0.01, respectively.

**Table S3.** Assuming DNA and RNA bases have similar hydration, we compared hyrdation levels observed in crystallographic data of unpaired RNA bases [1] to each other with zI test [2]. Water molecule per base was higher for purine bases (teal) than pyrimidine bases (brown) with statistical significance, implying that unpaired purine bases have higher entropic impact and are affected by temperature more.
