# Supplementary material for: Predicting stability of DNA bulge at mononucleotide microsatellite
Source: Nucleic Acids Res. 2021 Jul 26;49(14):7901–8. doi: 10.1093/nar/gkab616 (PMC8373066; doi:10.1093/nar/gkab616)
Supplement: gkab616_Supplemental_Files [file gkab616_supplemental_files.zip › Supplementary V2.pdf]

Section S1: Toehold Exchange Energy Measurement

The limitation of all conventional methods for DNA thermodynamics, such as melting curve analysis and calorimetry, is that they have to measure ΔG° of a whole DNA duplex. To infer $\Delta\Delta G^\circ$ of a variation, they compare ΔG° of a DNA duplex without any variation and ΔG° of a duplex with a variation. However, this is not an ideal approach because it involves a subtraction between two large numbers to get a small answer that makes it prone to errors.

To avoid such vulnerability while inferring $\Delta\Delta G^\circ$, we used Toehold Exchange Energy Measurement (TEEM) which utilizes toehold exchange reactions and measures their $\Delta G^\circ_{\mathrm{rxn}}$ from fluorescence. TEEM compares $\Delta G^\circ_{\mathrm{rxn}}$ of two toehold exchange reactions instead of ΔG° of two whole duplexes. Because $\Delta G^\circ_{\mathrm{rxn}}$ is a difference between ΔG° sum of products and that of reactants, most parts of duplexes automatically cancels out due to overlapping regions in TEEM. As a result, TEEM reduces sizes of numbers in the actual calculation and leaves much less room for an error. For example, a subtraction between ΔG° of two whole duplexes $62 - 60 = 2$ kcal/mol can be reduced to $3 - 1 = 2$ kcal/mol by TEEM.

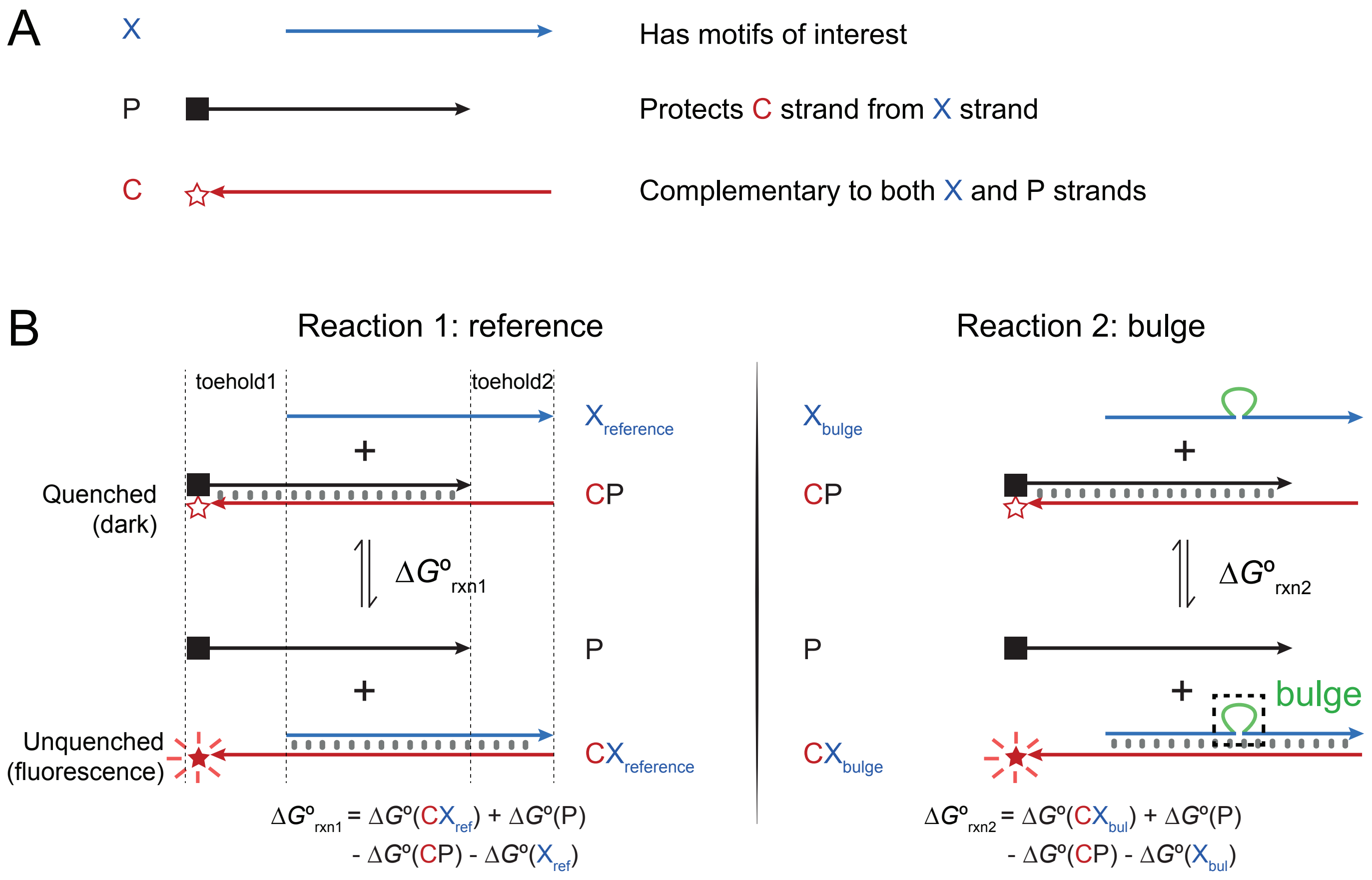

$$\Delta\Delta G^\circ_{\mathrm{bul}} = \Delta G^\circ(\mathrm{CX_{bul}}) - \Delta G^\circ(\mathrm{CX_{ref}})$$

$$\begin{aligned}\Delta G^\circ_{\mathrm{rxn2}} - \Delta G^\circ_{\mathrm{rxn1}} &= \Delta G^\circ(\mathrm{CX_{bul}}) - \Delta G^\circ(\mathrm{X_{bul}}) - \Delta G^\circ(\mathrm{CX_{ref}}) + \Delta G^\circ(\mathrm{X_{ref}}) \\ &= \Delta\Delta G^\circ_{\mathrm{bul}} - \Delta G^\circ(\mathrm{X_{bul}}) + \Delta G^\circ(\mathrm{X_{ref}}) \approx \Delta\Delta G^\circ_{\mathrm{bul}}\end{aligned}$$

FIG. S1-1: **(A)** Roles of three types of strands in TEEM. **(B)** Because the only difference between Reaction 1 and 2 is the presence of a bulge in X oligo, subtracting two ΔG° of reactions cancels out and leaves $\Delta\Delta G^\circ_{\mathrm{bul}}$, $\Delta G^\circ(\mathrm{X_{bul}})$, and $\Delta G^\circ(\mathrm{X_{ref}})$. Folding energies of both $\Delta G^\circ(\mathrm{X_{bul}})$ and $\Delta G^\circ(\mathrm{X_{ref}})$ can be considered 0 kcal/mol because X oligos are designed not to have any secondary structures, and any other contributors to ΔG° should be canceled out due to high similarity between the sequences. Therefore, the gap between two ΔG° of reactions approximates to $\Delta\Delta G^\circ_{\mathrm{bul}}$.

Section S2: Raw $\Delta\Delta G^\circ$ of Non-, 2-, and 3-slide Bulges

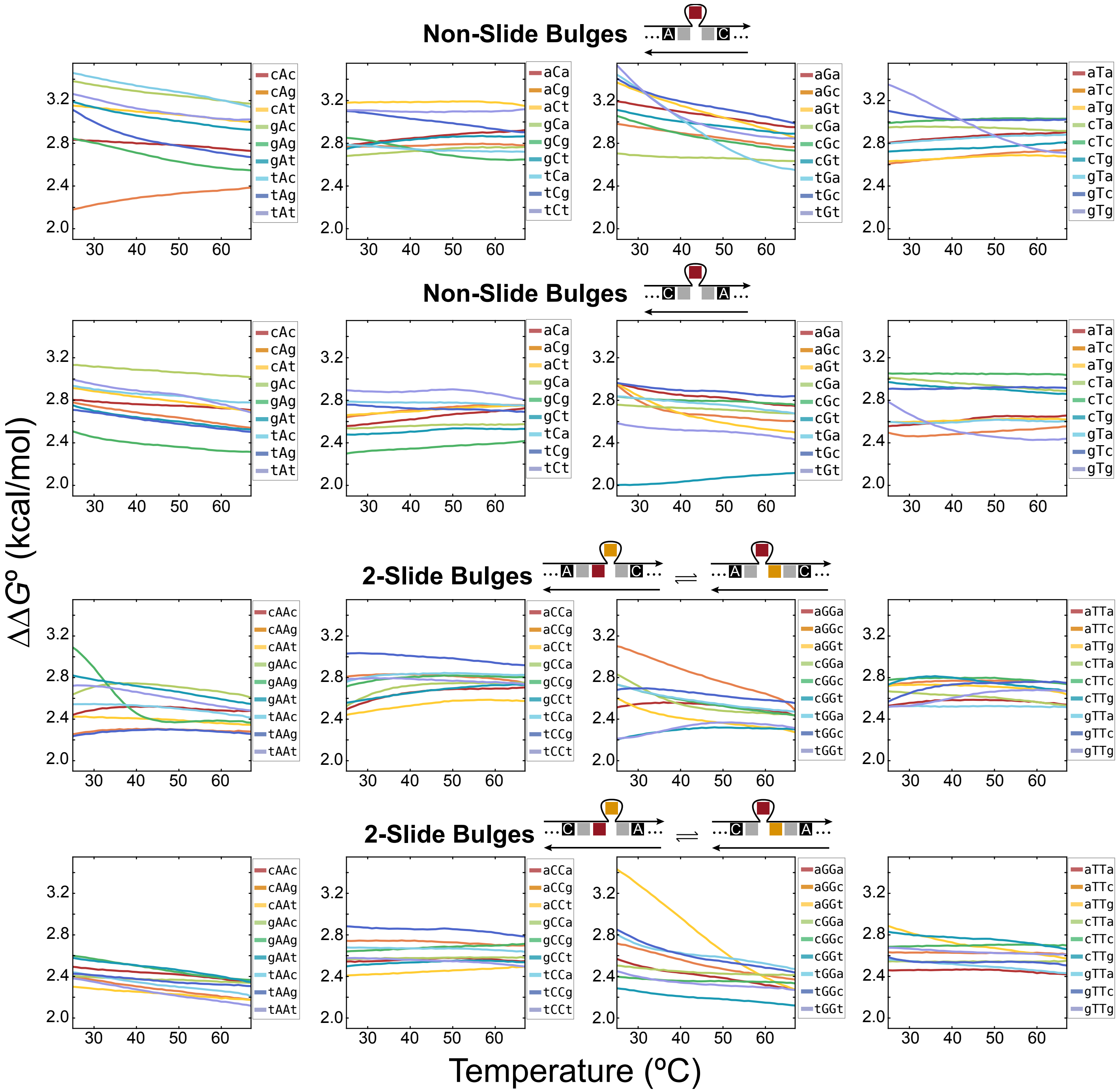

FIG. S2-1: Raw $\Delta\Delta G^\circ$ of non- and 2-slide bulges. There are two $\Delta\Delta G^\circ$ for each motif because we measured every $\Delta\Delta G^\circ$ twice with different NNN, A/C and C/A. $\Delta\Delta G^\circ$ 6 non-slide bulges with A/C as NNN out of all 144 bulges motifs were adopted from our previous publication [30]. Bulges that showed different results within each group always included G base in their sequence, possibly because low mismatch penalty of G allowed forming structures other than the intended bulges, depending on the surrounding sequences.

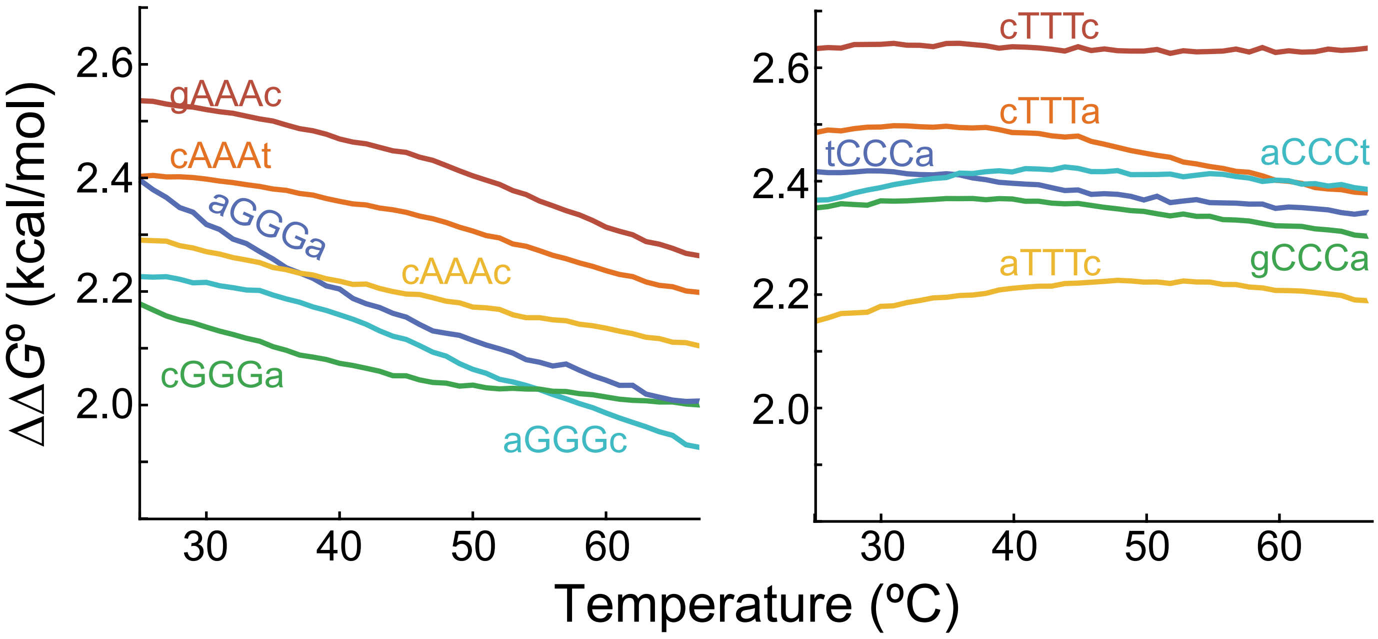

FIG. S2-2: Raw $\Delta\Delta G^{\circ}$ of selected 3-slide bulges.

Section S3: Derivation of the Prediction Equation

According to the canonical partition function of a discrete system, $\Delta\Delta G^\circ(\text{cTTc})$ can be expressed as

$$\Delta\Delta G^\circ(\text{cTTc}) = -RT \cdot \ln\left(e^{-\frac{\Delta\Delta G^\circ(\text{cTt})}{RT}} + e^{-\frac{\Delta\Delta G^\circ(\text{tTc})}{RT}}\right) \tag{1}$$

Rearranging equation 1 results in

$$e^{-\frac{\Delta\Delta G^\circ(\text{cTTc})}{RT}} = e^{-\frac{\Delta\Delta G^\circ(\text{cTt})}{RT}} + e^{-\frac{\Delta\Delta G^\circ(\text{tTc})}{RT}} \tag{2}$$

In a similar manner,

$$\Delta\Delta G^\circ(\text{cTTTc}) = -RT \cdot \ln\left(e^{-\frac{\Delta\Delta G^\circ(\text{cTt})}{RT}} + e^{-\frac{\Delta\Delta G^\circ(\text{tTc})}{RT}} + e^{-\frac{\Delta\Delta G^\circ(\text{tTt})}{RT}}\right)$$

$$e^{-\frac{\Delta\Delta G^\circ(\text{cTTTc})}{RT}} = e^{-\frac{\Delta\Delta G^\circ(\text{cTt})}{RT}} + e^{-\frac{\Delta\Delta G^\circ(\text{tTc})}{RT}} + e^{-\frac{\Delta\Delta G^\circ(\text{tTt})}{RT}} \tag{3}$$

Subtracting equation 2 from equation 3 yields

$$e^{-\frac{\Delta\Delta G^\circ(\text{tTt})}{RT}} = e^{-\frac{\Delta\Delta G^\circ(\text{cTTTc})}{RT}} - e^{-\frac{\Delta\Delta G^\circ(\text{cTTc})}{RT}}$$

This value directly fills in the following prediction equation.

$$\Delta\Delta G^\circ(\text{cT}^N\text{c}) = -RT \cdot \ln\left(e^{-\frac{\Delta\Delta G^\circ(\text{cTTc})}{RT}} + (\text{N-2}) \cdot e^{-\frac{\Delta\Delta G^\circ(\text{tTt})}{RT}}\right)$$

Note that tTt triplet state is universal for all T sliding bulges and can be combined with the other 2-slide bulges to predict different sliding bulges.

Section S4: Non-slide Bulge $\Delta\Delta G^\circ$ in the Literature

Although non-slide bulges have been studied in the past, there are not many papers covering all 36 motifs. SantaLucia *et al.* included $\Delta\Delta G^\circ$ values of non-slide bulges in a paper about energies of various DNA motifs, but it assigned only 4, 4.5, or 5 kcal/mol to all 36 bulges [1]. Moreover, no experimental data was disclosed to support this approximation. Another paper by Tanaka *et al.* did actually measure the energies of the bulges, but the error-prone nature of melting curve analysis resulted in large errors [2].

All old methods including the melting curve analysis calculate $\Delta\Delta G^\circ$ of a motif of interest by subtracting ΔG° of a duplex without the motif from ΔG° of a duplex with the motif. However, this approach is vulnerable to errors because it involves a subtraction between two large numbers to get a small answer. TEEM compares $\Delta G^\circ_{\mathrm{rxn}}$ of two toehold exchange reactions instead of the whole DNA duplexes to achieve higher accuracy.

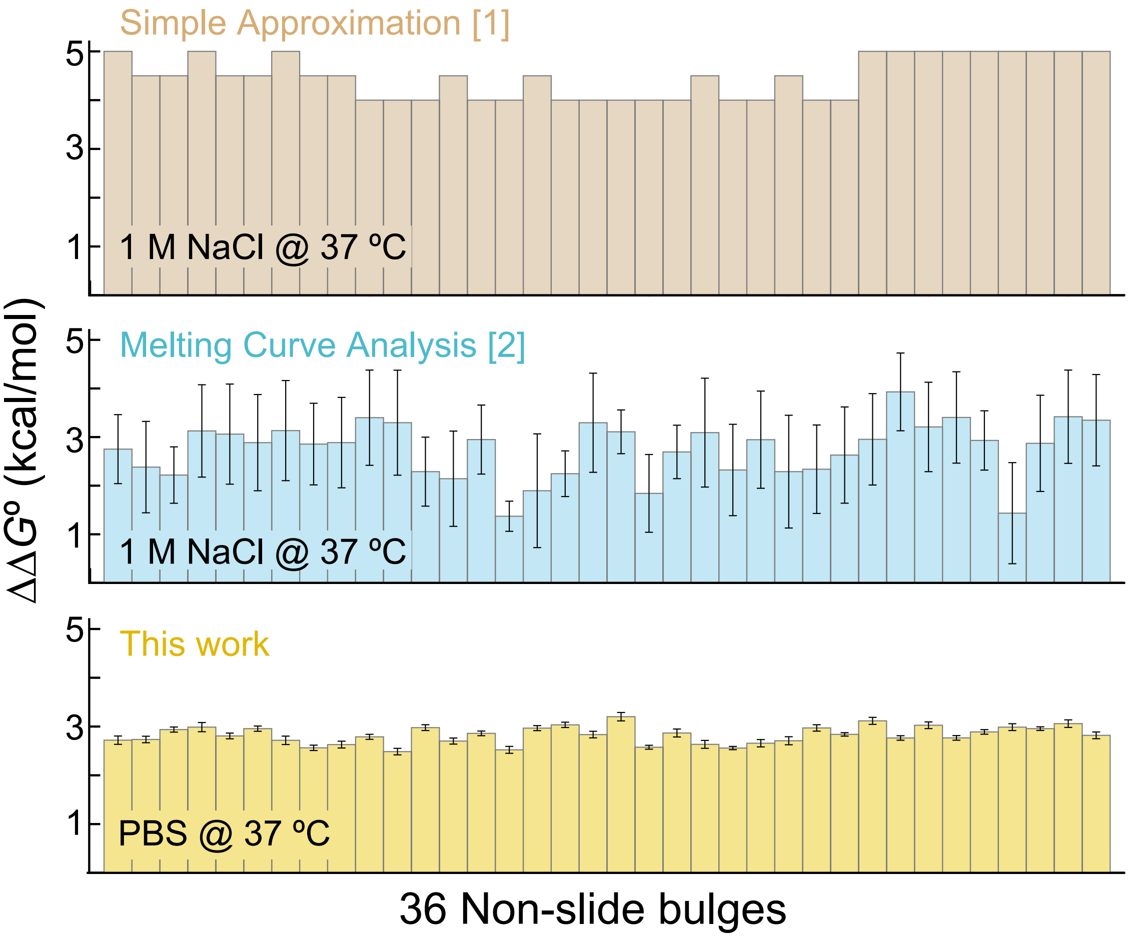

FIG. S4-1: Comparison between non-slide bulge $\Delta\Delta G^\circ$ in the literature and this study. The first plot shows a simple approximation which uses only 3 values for all bulges, 4, 4.5 or 5 kcal/mol, without any experimental data [1]. The second plot summarizes the results of melting curve analyses, but their error ranges are significant [2]. In addition, the concentration of cations in both of them was 1 M which is far from biological salinity, whereas TEEM measured non-slide bulge $\Delta\Delta G^\circ$ at PBS (pH 7.4, 150 mM cation), which is isotonic to human cells, with much smaller error range. The bulges were sorted in alphabetical order, and the error bars indicate 1 standard deviation.

[1] SantaLucia, J., Jr., & Hicks, D. The thermodynamics of DNA structural motifs. Annu. Rev. Biophys. Biomol. Struct 33, 415-440 (2004).

[2] Tanaka, F. et al. Thermodynamic Parameters Based on a Nearest-Neighbor Model for DNA Sequences with a Single-Bulge Loop. Biochemistry 43, 7143-7150 (2004).

Section S5: $\Delta\Delta G^\circ$ Deviation from NNN Choices

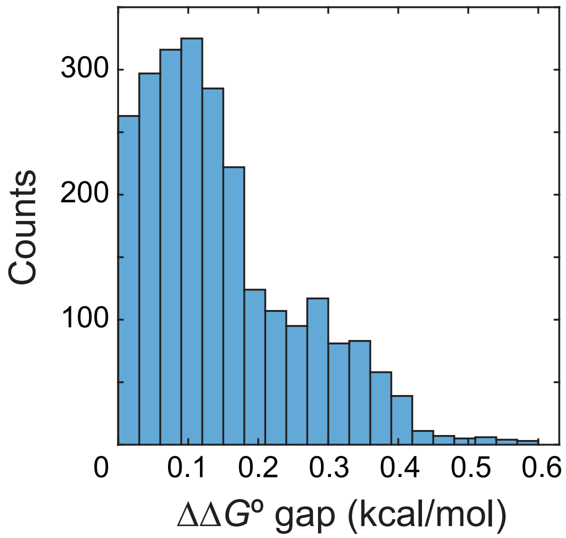

FIG. S5-1: Summary of all $\Delta\Delta G^\circ$ gap between true $\Delta\Delta G^\circ$ and the representative $\Delta\Delta G^\circ$. We first calculated absolute differences between all measured $\Delta\Delta G^\circ$ from Figure 2A and their corresponding representative $\Delta\Delta G^\circ$, which is the mean of two $\Delta\Delta G^\circ$ sets with either A/C or C/A as the NNN. Because the only difference is the NNN, this demonstrates the size of an error when actual NNN is different from the representative NNN. The resulting 2064 values had a mean of 0.15 kcal/mol and a standard deviation of 0.008 kcal/mol.
